# Unlocking the HOX: Homeobox Genes as Regulators of Hematopoietic Development

**DOI:** 10.3390/ijms27073285

**Published:** 2026-04-04

**Authors:** Daniel A. Moyer, Anika M. Henning, Kay L. Medina

**Affiliations:** 1 Mayo Clinic Graduate School of Biomedical Sciences, Mayo Clinic, Rochester, MN 55905, USA; moyer.daniel2@mayo.edu; 2 Department of Immunology, Mayo Clinic, Rochester, MN 55905, USA; henning.anika@mayo.edu; 3 Department of Hematology, Mayo Clinic, Rochester, MN 55905, USA

**Keywords:** homeobox, hematopoiesis, transcription factor, hematopoietic stem cell, lymphoid development, B cells

## Abstract

Homeobox (HOX) transcription factors are encoded within highly organized loci expressed along an anterior–posterior axis through embryogenesis and in a pleiotropic manner in hematopoiesis. HOX expression has been exhaustively studied in the context of oncogenesis and malignancy, but the compensatory substitution of HOX paralogs makes mechanistic annotation in steady-state hematopoiesis challenging. Despite this, HOX genes reflect numerous non-redundant roles in healthy hematopoiesis including HSC self-renewal, development, lymphopoiesis, myelopoiesis, and erythropoiesis. Here, we review historical and current insights into HOX functions in steady-state hematopoiesis and highlight unexplored avenues in their biology that could further elucidate their significance to hematopoietic homeostasis.

## 1. Background

**Figure 1:**
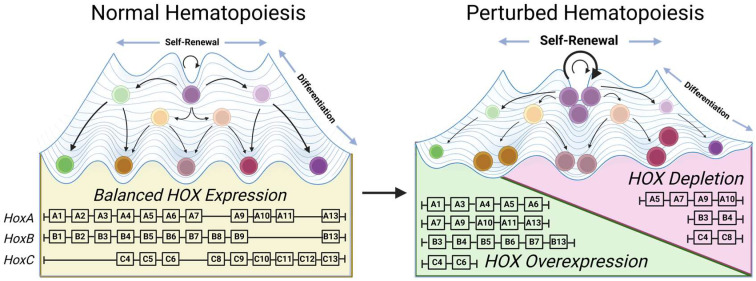
Hematopoietic perturbations arise with imbalanced HOX expression. Whether by gain or loss of function in HOX gene expression, selected loci affect the hematopoietic landscape, altering the hematopoietic maintenance of balanced self-renewal versus differentiation. Created in BioRender. Moyer, D. (2026) https://BioRender.com/q2kc3lr accessed on 29 January 2026.

Hematopoiesis is maintained throughout life by self-renewing hematopoietic stem cells (HSCs) that give rise to diverse blood progeny through highly ordered and regulated differentiation processes [1,2,3]. Errors affecting hematopoiesis have increased in prevalence with the extending of the human lifespan and have been attributed to an imbalance of self-renewal and differentiation [3,4]. Niche constrained cellular populations, such as hematopoietic progenitors, benefit from selecting self-renewal superiority, outcompeting those which prioritize differentiation potential [5,6]. This can be plainly observed with the increased incidence of malignancies and non-malignant clonal processes such as clonal hematopoiesis of indeterminate potential (CHIP) and myelodysplastic syndrome (MDS), along with the decrease in lineage-balanced hematopoietic stem cells in older adults [5,7,8,9]. Mechanisms that affect the balance of self-renewal and differentiation potential have been interrogated on numerous levels and have highlighted homeobox (HOX) transcription factors in these disorders (Figure 1) [5,10,11,12,13].

Homeosis, as proposed by Bateson, is the transformation of one structure of the body into homologous structures of other body segments [14]. HOX genes represent some of the earliest discovered homeotic alleles which specify the spatial organization of organisms, sharing a common DNA sequence element, the homeobox [14,15]. In Drosophila, where these loci were first described, HOX genes are arranged into ANT-C and BX-C loci [15]. Specific perturbations result in dysmorphic phenotypes which initially defined the HOX code, a set of rules which governs expression and patterning encoded by HOX genes and has been extensively reviewed [16,17,18,19,20]. When the homeobox was first identified, it was lauded as a unique motif for segmentation genes, but this domain has since been found in a wide range of eukaryotic regulatory genes [21,22].

Homeobox domains have been annotated in 241 human protein-coding genes and 277 orthologous mouse genes [21]. Indeed, homeodomains are not the hallmark of genes playing any singular role in development [18]. Since the discovery and cloning of HOX loci in the 1970s, it has been found that most eukaryotic organisms contain HOX genes; however, HOX loci are rarer among organisms [23]. Contrary to genomic entropy anticipated with evolutionary time, HOX genes have generally increased in genomic organization in higher-order organisms [24]. The mechanisms leading to HOX concentration in specific genetic loci are heavily debated and reviewed exhaustively elsewhere [22,24,25,26]. Evolutionary duplication has led to the rise of four HOX loci in most vertebrates (with the known exceptions of teleost and lamprey) each encoded on separate chromosomes, and in humans and mice, they contain 39 paralogs [23]. HOX genes represent a variety of paradigms in developmental, evolutionary, and genomic biology, with there being abundant reviews on each of these topics [18,19,22,25,26,27,28,29]. The temporal expression and lineage specificity of genes encoded in the HOXA-C loci in bone marrow during hematopoietic differentiation, as well as their oncogenic potential, have previously been reviewed [30]. In some but not all cases, the functions of HOX proteins in malignancies recapitulate homeostatic HOX biology and have aided in furthering our understanding of these loci [31].

This review will delve into the historical and recent literature regarding homeobox transcription factors in normal hematopoietic biology, outlining well-treaded territory as well as open questions in the field. We will concentrate on primary data from murine and human models of hematopoiesis, focusing on ex vivo culture systems and in vivo models. In vitro cell line and primary cancer studies will be minimally used to differentiate malignant from homeostatic HOX biology and to draw awareness to understudied loci and mechanisms in healthy systems. We hope that by coalescing the most relevant data, we can distinguish homeostatic from malignant and/or artificially induced HOX biology. Finally, by highlighting caveats inherent to the interpretation of overexpression systems to normal biologic function, we can be more intentional with the design of experimental approaches in the future.

## 2. Studies of HOX Genes in Hematopoiesis

Difficulty in studying homeobox genes is, in part, attributed to divergent findings from overexpression and knockout studies (Figure 1). The overexpression of many homeobox genes yields drastic alterations in cellular proliferation and improved self-renewal potential, while most knockouts show minimal effects (Figure 1). *Hoxa9*, *Hoxb3*, and *Hoxb4* are the most highly expressed homeobox genes in hematopoietic stem and progenitor cells (HSPCs), and have both knockout and overexpression phenotypes [32,33,34,35]. The relative expression levels of all homeobox genes remain low throughout hematopoietic differentiation, as established by early qPCR studies [36]. Additionally, hematopoietic progenitors are a small fraction of cells contained within relevant tissues, and ex vivo culture often changes their multipotency and self-renewal ability [37]. Changes to HOX expression are often seen in ex vivo culture systems and further complicate elucidating true homeobox biology [38].

HOX proteins contain very similar but non-identical homeodomain binding sites, requiring cofactors to carry out transcriptional control [29]. Numerous homeobox proteins can often be found in the same promoter sites and can play redundant roles to paralogs or members of the same loci [39]. One of the many issues in knockout studies of homeobox genes is the lack of understanding in these redundancies. Thus, the issue of compensatory mechanisms creates further biological confusion. Additionally, overexpression models of HOX proteins can illuminate the broad spectrum of genes which have homeodomain-containing regulatory regions, but the biological significance of these in homeostatic hematopoietic biology is unclear. The increased accessibility of proteomic approaches to understanding protein–protein interactions at specific regulatory sites will be essential in clarifying these relationships.

To date, roles for the homeobox loci A, B, and C have been established in studies utilizing numerous cell lines, as well as ex vivo and in vivo studies of murine or human primary hematopoietic cells (Table 1), while homeobox D has shown little significance [36]. The in vitro manipulation of select Hoxd paralogs in human embryonic or induced pluripotent stem cells demonstrated varied results which have not been translated to other model systems [40]. While Hoxd loci deserve more rigorous investigation, for the purposes of this review, we focus on studies of homeobox A, B, and C paralogs which have shown regulatory significance in many aspects of hematopoiesis.

### 2.1. Homeobox A

The Hoxa locus, with great emphasis on *Hoxa9*, has been studied extensively for its role in acute myeloid leukemia (AML) and oncogenesis [13]. Homeobox genes can lead to tumor progression, as seen with transgenic mouse models, where overexpression can block terminal differentiation and augment self-renewal ability [41]. The constitutive overexpression of all Hoxa loci except *Hoxa2* and *Hoxa5* leads to the blockade or delay of hematopoietic differentiation [41]. The ability of homeoproteins like Hoxa9 to serve as activators of genes that promote survival and repressors of apoptotic genes further underscores their oncogenic value [42,43]. Murine Hoxa9 overexpression has been found to enhance HSC expansion and myeloid progenitor proliferation and lead to leukemias with long latency [44,45]. Additionally, this overexpression can inhibit pre-B cell differentiation but does not seem to impact T cell development [35]. Inducible models of Hoxa9 expression have yielded great insights into its regulatory roles in cell cycle kinetics, differentiation, and the maintenance of stemness [44,45,46,47]. It is to be noted that Hoxa9 alone is not sufficient to dedifferentiate a cell or return it to a more progenitor-like state [48]. Hoxa9 is also the most studied in hematopoiesis due to the lack of functional redundancies and its relatively high expression in HSCs among the HOX loci [49]. Hoxa9 expression is high in HSCs and is downregulated through differentiation [50]. Hoxa9-deficient mice demonstrate a shifted HSC compartment with deficiencies in lymphopoiesis, erythropoiesis, and thrombopoiesis and a reduced cellularity of the spleen and thymus [51,52]. When transplanted or subject to sublethal irradiation, Hoxa9-deficient murine bone marrow produces fewer lymphocytes than its wildtype counterparts [50]. This intrinsic deficiency is recapitulated ex vivo, showing fewer high-proliferative potential colonies and the delayed development of myeloid-committed progenitors [50]. These phenotypes were found to be corrected upon the reintroduction of Hoxa9 expression [50]. Hoxa9 has also been attributed to maintaining quiescence and self-renewal potential to HSCs [53].

There are limited studies examining the functional significance of the other Hoxa loci. This is interesting as many Hoxa paralogs show higher relative expression levels than those of B or C in hematopoietic cells [36]. HOX gene dosage appears important as the haploinsufficiency of the full Hoxa locus resulted in mild disruption, while full deletion significantly perturbed hematopoiesis [54,55]. Exogenous Hoxa3 delivered to murine hematopoietic progenitor cells improved their proliferation, increased the production of CFU-G, and reduced the amount of CFU-M [56]. Hoxa4 overexpression was shown ex vivo to expand HSCs and primitive progenitors while still giving rise to normal proportions of myeloid and lymphoid progeny [57]. In vivo, the constitutive expression of Hoxa4 led to the preferential expansion of B cell progenitors [57]. Treating human bone marrow with antisense oligonucleotides to HOXA5 inhibited myelopoiesis and increased the generation of erythroid progenitors, while constitutive overexpression performed by a different group resulted in the inverse [58,59]. Hoxa6 overexpression demonstrated enhanced proliferation and self-renewal in the colony formation of murine HSPCs [60]. Medial HOXA genes are important for HSC identity and often fail to be upregulated when deriving HSPCs from human embryonic stem cells (hESCs) [61]. The knockdown of HOXA7 in human fetal liver-derived HSPCs disrupted function and transcriptomic signatures similar to hESC-derived HSPCs [61]. Murine Hoxa7-deficiency was found to reduce megakaryocyte–erythroid progenitor (MEP) numbers in vivo [62]. Conversely, the conditional overexpression of Hoxa7 in largely unfractionated murine bone marrow cells generated immortalized MEPs dependent on thrombopoietin (TPO) for survival (Hoxa7-TPO) [63]. The overexpression of HOXA10 in human CD34+ hematopoietic cells reduced BFU-E colony formation but improved the self-renewal of clonal progenitor cells [64]. Hoxa10 overexpression in murine HSPCs demonstrated similar progenitor expansion with dysregulated myeloid, erythroid, megakaryocytic, and lymphoid differentiation [65,66]. The loss of Hoxa10 expression in mice impaired megakaryopoiesis, erythropoiesis, and recovery from induced thrombocytopenia [67]. Early microarray data demonstrated that the gene sets regulated by Hoxa9 and Hoxa10 are highly similar [68].

### 2.2. Homeobox B

Hoxb genes have been found to play large roles in HSC proliferation and differentiation, though they are not critical for the specification of HSCs or definitive hematopoiesis [33,69,70]. *Hoxb3* and *Hoxb4* are the highest-expressed Hoxb loci in hematopoietic stem cells [33]. When Hoxa9, Hoxb3, and Hoxb4 were knocked out in concert, they were found to cooperatively regulate HSC biology [71]. Deficiencies in Hoxb3 and Hoxb4 were found to reduce the fetal liver stem cell pool but are not required for their development [72]. Hoxb loci, especially *Hoxb3*, play critical roles in lymphopoiesis [73]. Hoxb3 is expressed in quiescent HSCs, and expression is maintained through erythropoiesis and granulopoiesis [33]. The overexpression of Hoxb3 results in defects in T and B lymphoid development and significant increases in the myeloid progenitor pool [74]. Interestingly, Hoxb3-deficient mice also show reduced B cell pools starting at 3 months of age and significantly perturbed B cell development by 6 months [73]. Despite this, the loss of Hoxb3 expression is not necessary for lymphoid maturation, but the downregulation of this gene is critical for steady-state hematopoiesis [72,73].

Hoxb4 is normally expressed in human and murine HSPCs in bone marrow and the fetal liver, with overexpression resulting in the expansion of these compartments both ex vivo and in vivo [75,76,77]. Hoxb4 overexpression systems have been used for the immortalization of numerous hematopoietic cell types [78]. Several studies have pointed to Hoxb4 as promoting HSC self-renewal; however, Hoxb4-deficient mice present with normal HSC numbers [34,79,80]. Hoxb4 has a unique 15-proline stretch which many have attributed to this paralog’s unique proliferative properties [81]. Numerous studies have pointed out its central role in cellular expansion, though limited evidence points to Hoxb4’s influence on differentiation potential [34,40,82].

Hoxb5 has been used in transgenic mouse models to label long-term HSCs and is turned off concomitantly with the transition to short-term HSCs [83,84,85]. Hoxb5+ HSCs are resilient to stress hematopoiesis and are considered the most primitive and important for maintaining hematopoiesis throughout life [86]. The forced expression of Hoxb5 can alter the differentiation potential of hematopoietic progenitors and alter cellular programming across lineages [87,88].

Hoxb6 is upregulated in early granulocytic, monocytic, and erythroid progenitors but is undetectable in human CD34+ hematopoietic cells [89,90]. In one study, the overexpression of Hoxb6 expanded HSCs and myeloid progenitors while impairing erythropoiesis and lymphopoiesis [91]. The results of this work point to the notion that Hoxb6 expression may promote granulocyte and monocyte development at the expense of other lineages, which is partially supported by early work in Hoxb6-deficient mice which demonstrate increased erythroid progenitors though no changes in other lineages [91,92].

Hoxb7 overexpression in HSPCs increased HPP-CFC, LTC-IC, CFU-GM, CFU-G, and CFU-M colony counts while not modifying the total number of HSPCs, suggesting preleukemic immortalization stimulus [93].

### 2.3. Homeobox C

Limited works exist regarding the significance of homeobox C genes in hematopoiesis and are mostly restricted to paralogs 4, 5, and 6. qPCRs of early hematopoietic progenitors have found limited expression patterns of HOXC with the upregulation of HOXC4 and HOXC6 but not HOXC5 through lymphoid differentiation [94]. Bijl et al. [95] found that HOXC4 transcripts were present in human HSCs (CD34+ CD38 low) and in terminally mature lymphoid cells. However, HOXC5 is only expressed at low levels throughout hematopoiesis, with higher expression found in select neoplastic cell lines [95]. HOXC6 expression is initiated in prothymocyte and pre-pro-B cell stages and maintained in mature cells [95]. HOXC4 overexpressed in human CD34+ HSCs expanded CFU-GM, BFU-E, and LTC-IC but did not cause myeloid colony skewing [96]. HOXC6 overexpressed in HSPCs and transplanted into murine recipients showed an enhanced repopulation capacity, myeloid differentiation, and expansion of granulocyte macrophage/common myeloid progenitors (GMPs/CMPs) [97]. Ex vivo, HOXC6 overexpressing HSPCs demonstrated improved colony formation in comparison to wildtype counterparts [97].

HOXC8 is expressed at varied levels in human peripheral blood mononuclear cells (PBMCs) but in a less lineage restricted fashion [94]. Shimamoto et al. [98] explored this in a transgenic knockout mouse model of Hoxc8, finding significant reductions in BFU-E and CFU-GM but normal peripheral blood counts. Interestingly, Hoxc8-deficient hematopoietic cells were said to perform like wildtype controls ex vivo, so the authors posited that there could be an extrinsic defect they were not accounting for in the mice [98].

The extracellular expression of homeobox genes can influence cellular activity, as demonstrated by Auvray et al. [96], who co-cultured Hoxc4-producing stromal cells with human CD34+ HSCs. They found that extrinsic Hoxc4 expression expanded human HSCs 3–6 times ex vivo and significantly improved in vivo engraftment ability [96]. The ability of homeobox proteins to cross the plasma membrane is a powerful yet underutilized system for “noninvasive” overexpression. When comparing the transcriptomes of Hoxc4-exposed human CD34+ HSCs to those genetically engineered to overexpress Hoxb4, they found that both HOX proteins regulate the same set of genes to promote proliferation [96].

Work by Park et al. [99] suggests that Hoxc4 directs regulatory control over activation-induced cytidine deaminase (AID) expression. Hoxc4 deletion perturbs class switch recombination (CSR) and somatic hypermutation (SHM), processes critical for the development and diversity of germinal center B cells (GC B cells) [99]. This group identified a binding site of Hoxc4 with Oct in the murine *Aicda* and human *AICDA* promoter, which synergized with conserved binding sites for critical transcription factors Sp1, Sp3, and NF-kB [99]. Hoxc4 was found to be expressed in GC B cells preferentially. GC B cell activation by CD40-CD40L engagement, LPS, or IL-4 resulted in Hoxc4 upregulation. Furthermore, Hoxc4-deficient GC B cells showed lower AID expression, resulting in impaired CSR and SHM, which could be fully restored by overexpressing AID [99]. This paper illuminated a potential non-redundant role for Hoxc4; however, due to editorial concerns, the results should be interpreted with caution and repeated for clarity.

**Table 1:** Murine models with engineered HOX loci used for hematopoietic studies.

Mouse Strain	Hematopoietic Phenotype	References
Hoxa^+/−^ (full Hoxa locus haploinsufficiency)	Increased primitive HSCs, reduced mature B cells with age, larger proportion of bone marrow-resident Mac1/Gr1 neutrophils, less competitive in transplantation	[54] Lebert-Ghali et al. 2010
Hoxa^fl/fl^ Mx1-Cre (inducible deletion of full Hoxa loci)	Reduced WBCs, RBCs, and platelets; reduced cellularity in all hematopoietic organs assessed; severe decrease in B cells; reduced HSPCs; reduced HSC proliferative potential but more cycling in vivo; less competitive in transplantation; no repopulation of secondary recipients	[55] Lebert-Ghali et al. 2016
Hoxa7^−/−^	Megakaryocyte/erythroid progenitor reductions	[62] So et al. 2004
Hoxa9^−/−^	Shifted HSC compartment; deficiencies in lymphopoiesis, erythropoiesis, and thrombopoiesis; reduced thymic and splenic cellularity; poor response to sublethal irradiation; poor reconstitution efficacy upon transplantation, maintenance of quiescence and self-renewal potential	[35,51,52] Thorsteinsdottir et al. 2002; Izon et al. 1998; Lawrence et al. 1997
Hoxa9^−/−^Flt3L^−/−^	Increased myeloid-biased HSCs; decreased lymphoid-primed multipotent progenitors, all-lymphoid progenitors, B-primed lymphoid progenitors, and B cells; reduced thymic cellularity	[100] Gwin et al. 2013
Hoxa9^−/−^β-catenin^fl/fl^ Rosa-Cre^ER^	Reduced numbers of long- and short-term HSCs and common myeloid, megakaryocyte/erythroid, and granulocyte/monocyte progenitors; reduced LTC-IC performance; increased cycling and DNA damage	[53] Lynch et al. 2024
Hoxa9^−/−^Hoxb3^−/−^Hoxb4^−/−^	Reduced spleen cellularity, increased HSCs, reduced repopulation capacity (similar to Hoxa9^−/−^)	[65] Magnusson et al. 2007
Hoxa10^−/−^	Deficiencies in megakaryopoiesis and erythropoiesis	[67] Konieczna et al. 2016
Hoxb3^−/−^	Impaired B lymphopoiesis, reduced bone marrow cellularity	[73] Ko et al. 2007
Hoxb3^−/−^Hoxb4^−/−^	Reduced repopulation capacity, reduction in HSPC pool, reduced hematopoietic organ cellularity, slower cell cycle kinetics in response to hematopoietic stress	[72] Bjürnsson et al. 2003
Hoxb4^−/−^	Reduced spleen and bone marrow cellularity, reduced RBCs and hemoglobin, defects following bone marrow transplantation due to impaired proliferative capacity	[79] Brun et al. 2004
Hoxb5^fl/fl^-Cre^ERT2^-Tomato	Fluorescent tracking of LT-HSCs with Hoxb5 expression resulting in Tomato expression	[83,85] Kucinski et al. 2024; Xiang et al. 2025
Hoxb5^fl/fl^-BFP-Vav-Cre	Fluorescent tracking of Hoxb5 deletion by BFP expression, transient but no long-term lineage differentiation bias	[84] Zhao et al. 2024
Hoxb6^−/−^	Increased number of erythroid progenitors	[92] Kappen et al. 2000
Hoxc4^−/−^	Impaired class switch recombination and antibody response from B cells, in part due to lower AID expression	[99] Park et al. 2009
Hoxc8^−/−^	Reduced BFU-E and CFU-GM	[98] Shimamoto et al. 1999

Note: Only HOX-engineered mouse lines with documented use in hematopoietic studies listed, not reflective of all available models.

## 3. Genetic Regulation and Activity of HOX

Through the course of evolution, conventional homeobox loci have become highly ordered into compact clusters across four separate chromosomes in mammals with generally lesser structure through lower genetic phyla [27]. The generation of genomically compact loci is theorized to be genetically advantageous as this allows for enhancer sharing and autoregulatory feedback, as we discuss below [23]. HOX loci are expressed linearly in a 3′ to 5′ manner and, in addition to promoter/enhancer control, contain coexpressed miRNAs, retinoic acid response elements (RAREs), CTCF boundary sites, etc., that facilitate highly tunable expression in a context-dependent manner [101]. The peculiar regulatory principles surrounding HOX gene expression were established and termed the “HOX code”, reflecting their temporal and spatial distribution, as well as their posterior prevalence in developing organisms [101]. HOX genes are expressed in HSCs in a manner not dissimilar to early development, with lineage- and differentiation stage-restricted expression patterns [5]. The reductionist belief is that Hoxa genes are the most prevalent in myeloid cells, Hoxb in erythroid cells, and Hoxc in lymphoid cells, though current experimental data reflects higher complexity [31]. The majority of HOX expression in hematopoietic cells is retained in the most primitive HSCs and progenitor compartment and downregulated through fate commitment [5].

Studies on the mechanistic roles of HOX proteins within primary hematopoietic cells are limited and often result in immortalization ex vivo, which may obscure true biology [102]. Regardless, many but not all insights observed in cancer cell lines have been found to be biologically relevant to normal hematopoiesis. As discussed, the dosage of homeobox genes can play a large role in their biological function. Supraphysiologic HOX overexpression settings rarely reflect temporal expression patterns and may illuminate HOX biologic activity which is not illustrated by primary cells. Cancer cells will manipulate healthy biology to gain a selective survival advantage, so the repressive and non-advantageous roles of HOX genes will be biased against and less characterized. Hematopoietic differentiation is a complex process coincident with the activation or inactivation of multiple HOX genes. The loss of a single HOX gene presents a negligible or mild phenotype, while defects in the regulators of many homeobox genes present significant dysfunction. As transcriptional and epigenetic techniques have become more sensitive even to the level of the single cell, future studies of primary cells may be able to capture homeostatic hematopoietic biology with greater accuracy.

### 3.1. Cdx

Caudal-related homeobox genes (Cdx) comprise genes *Cdx1*, *Cdx2*, and *Cdx4* in humans and mice [103]. HOX gene activation depends on the coordinated activity of several signaling pathways [101]. It is suggested that Cdx transcription factors could be a core integrator of these signaling pathways in the growing posterior embryo [104]. Consensus binding sites for the three Cdx homologs are present in the promoters of multiple HOX genes [105]. Cdx genes have been found to interact with the pre-B cell leukemia transcription factor (Pbx) in non-hematopoietic settings, but their significance in HOX gene regulation is unclear [106]. Cdx factors may demonstrate functional redundancy in mammalian hematopoietic development. During embryonic hematopoiesis, differing Cdx loci have varied hematopoietic phenotypes which are worsened by compound knockouts [107]. The loss of Cdx1 does not affect hematopoietic development in zebrafish or mice but has been shown in mESC systems to have a mild phenotype [103,108].

In one study, *Cdx4* gene deletion in zebrafish suggested a requirement for definitive hematopoiesis which was not recapitulated in murine models [109,110]. Cdx4-deficient zebrafish progenitors lose their ability to generate hematopoietic cells, which can be rescued by the forced expression of HOX genes [109]. Cdx4 deficiency significantly perturbed the expression profiles of HOX genes in zebrafish. The germline or conditional loss of Cdx4 in mice had minimal effect on adult hematopoiesis [110]. Interestingly, Cdx4 overexpression rescued defective blood progenitor formation from mouse embryonic stem cells deficient in Mll [108]. Ex vivo B cell progenitor clonogenic potential was reduced in Cdx4-deficient animals, but no alteration in mature B cells was observed in vivo [110]. Interestingly, the ectopic pulsed expression of Cdx4 and Hoxb4 allowed for the generation of more representative HSCs from mESCs [111]. Hoxa9 repressed Cdx4 transcription in differentiating myeloid cells antagonizing activation by Hoxa10, both of which are facilitated by stage-specific tyrosine phosphorylation [112].

The published findings demonstrated a greater role for Cdx2 in mammalian hematopoiesis. The enforced expression of Cdx2 in murine hematopoietic progenitors resulted in the increased expression of Hoxa5, Hoxa7, Hoxa9, Hoxa10, Hoxb3, Hoxb6, and Hoxb8 [113]. When Cdx2 is mutated at its N-terminal domain, losing its ability to activate HOX genes, it also loses oncogenic potential in leukemogenesis studies [113]. Cdx2-deficient mESCs have impaired multipotent blood cell progenitor production in vitro, which was corroborated when injected into a healthy blastocyst, establishing a critical role in definitive hematopoiesis in vivo [108]. The early lethality of Cdx2-deficient mice led one group to generate Cdx1-deficient Tie2-Cre conditional Cdx2 knockout mice (CDX1/2 DKO), and they found that compared to their Cdx1-deficient littermates, Cdx2 is required for primitive hematopoiesis [107]. The molecular mechanisms of Cdx2 are attributed to the downregulation of critical hematopoietic TFs like Gata1, Gata2, Scl, Meis1, and Klf1 [107]. Cdx2 deficiency can be rescued by the exogenous expression of Scl [107].

### 3.2. PRC

Polycomb (Pc) genes were identified as repressors of HOX gene expression in Drosophila [14,114]. Similarly to HOX genes in this model, homeotic transformations resulted from Pc loci [14]. Pc proteins associate in two distinct multiprotein complexes, Polycomb repressor complexes (PRCs) 1 and 2, which repress genes through histone modifications, leading to chromatin compaction [115]. Canonical PRC1 leads to H2AK119ub, and PRC2 generates H3K27me3 repressive marks with non-canonical PRCs adding diversity to their regulatory repertoire. The correlation between H3K27me3 marks and inactive HOX clusters suggests the involvement of PcG in HOX gene silencing, and this is supported by genetic analyses in mice [116]. Mechanisms that direct PcG to HOX clusters produce global silencing during early development and secure proper spatial HOX gene expression at later developmental stages [117]. Recent data shows that PcG function relates to specific 3D chromatin configurations in the nucleus which appear important for HOX locus regulation [25]. The subnuclear localization of PRC components reveal PcG activity confined in distinct nuclear regions that colocalize with inactive HOX genes [25].

Hoxa9 has been proposed to recruit PRCs to repress cell cycle regulators such as p16ink4a [118]. The PRC1 component Bmi1 was shown to be important in the regulation of broader HOX expression in knockout studies of non-hematopoietic stem cells [119]. Two H3K27me3 demethylases which oppose PRC2 repression, Jmjd3 and Utx, have been shown to interact with HOX genes and modulate H3K27me3 levels at their promoters [120]. Utx has emerged as a promising HOX regulator given that experiments which alter its expression lead to HOX-like phenotypes [121]. Interestingly, Utx has been found to interact with Mll2, but the nature of this relationship is unclear [122].

### 3.3. Meis/Pbx

Myeloid ecotropic viral integration site (Meis) and Pbx are homeobox proteins which act as HOX cofactors, helping tune regulatory control over HOX-controlled processes [123,124]. Generally, HOX paralogs 1–10 bind to Pbx1, and HOX proteins 9–10 bind to Meis1 [125,126]. Pbx-HOX-Meis complexes have been observed, but their regulatory significance is not well defined [102,127]. Pbx1 and Meis1 are critically important for normal embryonic development as single deficiencies are embryonic-lethal [128,129]. Conditional or heterozygous knockout models have significant hematopoietic phenotypes and demonstrate considerable influence on homeobox regulation [130,131]. A patient with heterozygous loss of function in the MEIS1 gene was reported, presenting with congenital thrombocytopenia [132]. Many HOX proteins contain a conserved hexapeptide domain, N-terminal to the homeodomain, which facilitates interactions with Pbx family members [133]. Meis proteins also contain a homeodomain and are believed to be stabilized by interactions with Hox and Pbx proteins [125]. Berkes et al. [134] suggested that Pbx and Meis are pioneering TFs which can penetrate repressive chromatin and mark loci for activation by more sequence-specific TFs. Meis1 conditional knockout mice fail to perform megakaryopoiesis and show extensive hemorrhaging [135]. Meis2 was reported to play a role in definitive hematopoiesis in studies of knockout hESCs but had limited significance in megakaryopoiesis or platelet generation [136]. Mice deficient in Pbx2 do not show hematopoietic phenotypes, and the roles of Pbx3 and Meis3 in hematopoietic development have not been reported [137]. Commercially available inhibitors of HOX proteins, HOX/Pbx interactions, and Meis proteins have been developed for studies of malignant cells and may prove significant in future studies to delineate hematopoietic biology [138,139,140].

### 3.4. Mll

Mixed lineage leukemia (Mll) proteins are histone methyltransferases and global regulators of gene expression [141]. Mll1 is known to maintain HOX expression and is embryonic-lethal when deficient [142]. Genetically engineered Mll1-deficient HSPCs fail to generate adult lymphoid or myeloid cells, indicating a requirement for Mll1 expression at this primitive stage [143]. Indeed, Mll1 is essential for the development of HSCs and the transition from HSCs to MPPs [144,145]. Interestingly, the reactivation of a subset or even a singular HOX gene in Mll1-deficient HSPCs rescued hematopoietic colony frequency and growth, illustrating its intimate reliance on homeobox genes to exact its function [145]. Neither individual nor compound HOX gene knockouts or HOX cofactors recapitulate the profound hematopoietic phenotype observed in Mll1-mutant animals. Mll1 fusion protein-overexpressing cell lines demonstrate the promotion of 5′ HOX gene expression and Meis1 [146]. Interestingly, Hoxa9 and Hoxa7 knockout backgrounds inhibit the oncogenic potential of certain Mll fusion proteins [147].

### 3.5. Retinoic Acid Signaling

It was initially theorized that retinoic acid affects HOX gene expression based on the observation that teratogenic doses led to homeotic transformation [148]. This relationship was later demonstrated in hematopoietic progenitors as important for the maintenance of dormancy/quiescence [149]. In mESCs, it was observed that Hoxb and Hoxd loci were tightly compacted in chromatin, and upon retinoic acid-induced differentiation, the loci became more accessible [150]. In breast cancer cell lines, Hoxa5 expression was upregulated in response to RARβ induction, inducing apoptosis [151]. The responsible RARE is found at the 3′ end of *Hoxa5* [151]. Retinoic acid response elements have been identified in *Hoxa1*, *Hoxb1*, *Hoxb4*, *Hoxd4*, *Hoxb5*, *Hoxb6*, and *Hoxb8* [152,153,154,155,156,157,158,159,160]. Retinoic acid is known to sequentially activate HOX genes in a 3′ to 5′ fashion [161]. Medial HOX genes can be upregulated in hESC-derived HSPCs by treatment with retinoic acid, improving their proliferative and engrafting ability [61]. The inhibition of proximal HOX genes in concert with retinoic acid treatment inhibits posterior HOX upregulation [162].

### 3.6. β-Catenin/Wnt

Recent data have identified non-redundant roles of Wnt/β-catenin signaling in hematopoietic proliferation, survival, and differentiation and implicated the HOX gene family in their function [53]. β-catenin and Wnt signaling, often studied as a network, was initially reported to be dispensable for adult hematopoiesis based on conditional knockout mouse models [163]. A conditional knockout model activated at embryonic stages, however, found β-catenin to be essential in definitive embryonic hematopoiesis [164]. β-catenin activates *Hoxa10* and *Cdx4* in myeloid progenitors, interacting with their promoters [165]. β-catenin/Wnt signaling seems to determine developmental stages by which Hoxa9 and Meis1 activation induces leukemic transformation [166]. Hoxa10 was found to promote the expression of Fgf2 in myeloid progenitors in an overexpression model, inducing a PI3K-dependent increase in β-catenin [167]. Hoxa9 in concert with β-catenin regulates Prmt1 to affect stem cell quiescence and DNA replication dynamics [53]. In embryonic stem cells, it was found that BMP4 directs cells towards the hematopoietic fate by activating Wnt3a, which upregulates Cdx and HOX gene expression [168]. Wnt/β-catenin signaling pathways are altered in many AML fusion proteins where Hoxa9 biology is implicated [169].

### 3.7. Cytokines and Growth Factors

The expansion of cord blood-derived HSPCs in the presence of the early-acting cytokines SCL, FLT3L, and TPO results in the upregulation of Hoxb3, Hoxb4, and Hoxa9 and downregulation of Hoxb8 and Hoxa10 [170]. Thrombopoietin induces Hoxa9 nuclear transport in immature hematopoietic cells [171]. The tyrosine phosphorylation of Hoxa9 and Hoxa10 occurs in a Jak2-dependent manner in myeloid progenitors in response to differentiating cytokines [112]. Leukemias associated with high levels of Hoxa9 and Meis1 have a higher expression and mutational burden of Flt3 [172,173]. Functional synergy between Hoxa9 and Flt3 signaling in the regulation of B lymphopoiesis has been demonstrated by prior murine studies [100,174].

### 3.8. Non-Coding RNAs

HOX gene clusters contain antisense long non-coding RNAs (lncRNAs) which are known to play roles in HOX regulation [175]. In a comparative genomic analysis of human and mouse homeobox clusters, it was found that human homeobox genes are more highly enriched in lncRNAs than orthologous mouse loci [21]. This same study examined human cell line microarray data, finding long intergenic non-coding RNAs in HOX intergenic regions which appear to play regulatory roles in mechanisms not yet understood [21]. At least 30 of the 39 mammalian HOX 3′ UTRs have one or more conserved matches to vertebrate miRNAs [176].

miRNAs are short non-coding RNAs which repress gene activity by binding to the complementary 3′ UTRs of mRNAs [177]. This leads to the degradation or translational repression of the targeted locus [178]. miRNAs encoded in the intergenic regions of HOX loci have been shown to regulate HOX expression [179]. This proximity to their regulated loci helps ensure the context-dependent expression of the target, and regulation occurs simultaneously. In vertebrates, relevant miRNAs include miR-196, -10, and -615. miR-196 and its paralogs sit between Hox9 and Hox10 of the a, b, and c loci and are the most well studied [180]. miR-196b represses Hoxa9 and Meis1 expression, affecting myelopoiesis [181]. Outside of the miRNAs found interspersed in homeobox loci, Hoxa9 expression has been found to parallel miR-155 in knockout and overexpression models in murine bone marrow cells [182,183].

### 3.9. Cell Cycle Regulators

Cell cycle regulators enable HSCs to manage transitions from extended periods of quiescence to proliferation, enabling the generation of billions of blood cells daily [184]. The efficient regulation of this process is essential in preventing the exhaustion of HSCs and clonal populations with unregulated proliferation potential [7,185]. HOX proteins have been found to exact both enhancing and inhibitory regulation over the cell cycle through the transcriptional and directed regulation of cell cycle regulators [44,186]. Homeobox proteins have been found to be transiently associated with the cell cycle inhibitory protein geminin [187]. p21 has been identified as a Hoxa10 target and p53 for Hoxa5 [58,188]. Sufficient evidence has demonstrated that Hoxa9 can repress p16ink4a expression [47,186]. Cyclin-dependent kinase inhibitors, once thought to be checkpoints in cycling, are now known to interact directly with proteins and pathways central to differentiation and apoptosis [189,190].

### 3.10. Miscellaneous

A bioinformatic analysis of murine HOX genes demonstrated that around one-third produce alternative mRNA isoforms which are evolutionarily conserved in humans [21,23]. HOX proteins have also been shown to interact with functionally divergent proteins such as BTG2, Maf, and Smad1 [191,192,193]. Many HOX proteins interact with CBP/P300, blocking its histone acetyltransferase activity [194]. This level of regulation in HOX biology is poorly studied and more widespread than appreciated. Setbp1 overexpression confers self-renewal capability to myeloid progenitors in vitro, due to the upregulation of Hoxa9 and Hoxa10 [195]. TGF-β and bone morphogenic protein (BMP) inhibit the oncogenic potential of Hoxa9 through Smad4 [196]. The RNA interference of Rad21 suppresses hematopoietic self-renewal via the epigenetic regulation of *Hoxa7* and *Hoxa9*, reducing H3K27me3 at those loci [197]. Hoxa9 overexpression rescues heterozygous menin-deficient phenotypes including reduced WBC count and BM colony formation [198]. Trib1 modifies the transcriptional programs of Hoxa9 [199,200]. Work in human embryonic stem cells points to a critical role of HOXA9 in facilitating the transition to a HSC by the regulation of the NF-κB pathway [45]. Hoxa9 has been linked to JAK/STAT, MYC, and Bcl-2 signaling, which have been proposed to drive leukemogenesis [201]. An activation protein domain has been found for Hoxa10 and is conserved across other Hox10 paralogs, facilitating interaction with the Creb-binding protein (CBP), and it is homologous for PQ domains in E1a-interacting proteins [202]. This domain is not found in *Hoxa9*, furthering the regulatory distinction with *Hoxa10* [202].

## 4. Conclusions/Perspectives

Homeobox proteins have significant and non-redundant roles in hematopoiesis, but complicated compensatory regulatory schemes have limited our ability to understand individual components. With improved protein–DNA binding assays like CUT&RUN and CUT&TAG, we can now observe HOX interactions in limited numbers of progenitors with recent advancements providing single-cell resolution [53,203,204,205]. Genomic HOX binding sites are not especially rare and can be largely occupied during overexpression. Therefore, assessing HOX-DNA binding from primary hematopoietic tissues is necessary to determine physiologic binding sites. Prior studies have revealed slight differences in the binding specificities of HOX genes as well as alternate splice isoforms, the significance of which has not been connected to hematopoietic biology. Furthermore, HOX genes rarely act unilaterally and have low expression patterns. Many mechanistic insights into homeobox biology have been gleaned from overexpression models, where the majority of available DNA binding sites are occupied [206]. While overexpression studies can help overcome HOX redundancy, true biology is obscured by oncogenic roles. It is improbable that the only function of most HOX genes in hematopoiesis is to promote proliferation. Their potency as repressors of cell cycle inhibitors is understated and understudied. Comparisons of overall expression between model systems and primary tissue should be made to draw attention to model-specific disparities. It has never been easier to generate knockout mouse models, and exploring new HOX knockouts will clarify non-redundant hematopoietic roles. The protein–protein interactions of Hoxa9 have been elucidated in AML cell lines to demonstrate Hoxa9 binding partners important for oncogenic potential, but the question remains whether these are relevant for homeostatic hematopoiesis [207]. Low-input proteomic approaches for blotting and mass spectrometry have continually improved and should now allow for the resolution of protein interactions in scarce HSPCs. We come to the repeated finding that homeobox biology is context-dependent, so how should we interpret the findings from immortalized cells? The biology of homeobox overexpression systems generally shows consistent phenotypes regardless of the paralog. However, single-locus knockouts show few phenotypes and often are disregarded. Most of the foundational work on homeobox loci was done prior to the greater accessibility and affordability of more complicated omics techniques, so big and modern datasets on homeobox perturbations are needed.

For most studies of hematopoietic biology, conditional HOX expression systems are employed ex vivo or in cell lines [48,63,97]. From this work, we know that stage-specific HOX gene expression patterns are critical. Most murine models of HOX deletion are germline, though conditional murine models are becoming more accessible and represent an underexplored avenue in HOX biology. Based on our lab’s work, we suspect that homeobox genes have bigger roles in hematopoietic fate commitment than currently appreciated, which could be validated by conditional knockouts. While large transcriptomic hematopoietic atlases are publicly available, few investigations have focused on age-associated changes in HOX gene expression [208,209]. HOX genes make distinctive expressional changes in the transition from fetal to adult hematopoiesis, but are broader age-related shifts observed across the lifespan of an individual [209]? Many upstream regulators of HOX genes are implicated in hematopoietic aging, so could HOX genes act as secondary effectors? Furthermore, conditions of aged hematopoiesis like MDS and CHIP come with little information regarding HOX gene expression or regulation. Some studies point to the significance of HOX gene expression in hematopoietic cell self-renewal, but deletion in mice does not cause early hematopoietic failure [60,71]. Many open questions persist in adult hematopoiesis, and dissecting homeobox biology using modern approaches represents an area of discovery we hope to see reinvigorated.

## Data Availability

No new data were created or analyzed in this study. Data sharing is not applicable to this article.

## Abbreviations

The following abbreviations are used in this manuscript:

HSPC	Hematopoietic stem and progenitor cell
HOX	Homeobox
CHIP	Clonal hematopoiesis of indeterminate potential
MDS	Myelodysplastic syndrome
AML	Acute myeloid leukemia
hESC	Human embryonic stem cell
mESC	Mouse embryonic stem cell
MPP	Multipotent progenitor
LT-HSC	Long-term hematopoietic stem cell
HSC	Hematopoietic stem cell
TPO	Thrombopoietin
MEP	Megakaryocyte–erythroid progenitor
GMP	Granulocyte macrophage progenitor
CMP	Common myeloid progenitor
CLP	Common lymphoid progenitor
PBMC	Peripheral blood mononuclear cell
AID	Activation-induced cytidine deaminase
CSR	Class switch recombination
SHM	Somatic hypermutation
GC	Germinal center
PRC	Polycomb repressor complex
RARE	Retinoic acid response element
